# Optimization of mirtazapine loaded into mesoporous silica nanostructures via Box-Behnken design: *in-vitro* characterization and *in-vivo* assessment

**DOI:** 10.1080/10717544.2022.2075985

**Published:** 2022-05-25

**Authors:** Abeer A. Musallam, M. A. Mahdy, Hanan M. Elnahas, Reem A. Aldeeb

**Affiliations:** a Department of Pharmaceutics, College of Pharmaceutical Sciences and Drug Manufacturing, Misr University for Science and Technology, Giza, Egypt; b Department of Pharmaceutics, Faculty of Pharmacy, Zagazig University, Zagazig, Egypt

**Keywords:** Mirtazapine, mesoporous silica nanostructures, box-behnken design, oral bioavailability, *in-vivo* study

## Abstract

Employment of mesoporous silica nanostructures (MSNs) in the drug delivery field has shown a significant potential for improving the oral delivery of active pharmaceutical products with low solubility in water. Mirtazapine (MRT) is a tetracyclic antidepressant with poor water solubility (BCS Class II), which was recently approved as a potent drug used to treat severe depression. The principle of this research is to optimize the incorporation of Mirtazapine into MSNs to improve its aqueous solubility, loading efficiency, release performance, and subsequent bioavailability. The formulation was optimized by using of Box-Behnken Design, which allows simultaneous estimation of the impact of different types of silica (SBA-15, MCM-41, and Aluminate-MCM-41), a different drug to silica ratios (33.33%, 49.99%, and 66.66%), and different drug loading procedures (Incipient wetness, solvent evaporation, and solvent impregnation) on the MRT loading efficiency, aqueous solubility and dissolution rate. The optimized formula was achieved by loading MRT into SBA-15 at 33.33% drug ratio prepared by the incipient wetness method, which displayed a loading efficiency of 104.05%, water solubility of 0.2 mg/ml, and 100% dissolution rate after 30 min. The pharmacokinetic profile of the optimized formula was obtained by conducting the in*-vivo* study in rabbits which showed a marked improvement (2.14-fold) in oral bioavailability greater than plain MRT. The physicochemical parameters and morphology of the optimized formula were characterized by; gas adsorption manometry, scanning electron microscopy (SEM), polarized light microscopy (PLM), Fourier-transform infrared spectroscopy (FT-IR), differential scanning calorimetry (DSC), and X-ray powder diffraction (XRPD).

## 1. Introduction

Depression is a prevalent and dangerous health condition that interrupts an individual's ability to feel, think and behave normally. People suffering from depression seem to have a lousy mindset with a deep feeling of melancholy, anxiety, suicidal tendencies, interrupted sleep, and perhaps a diminished interest in formerly pleasurable activities for most of the day. It can also result in a vast number of mental and physical issues, as well as an impaired performance at work and home (Ph.D. Nemeroff et al., 2022). Regrettably, nearly 280 million individuals suffer from depression on a global scale. Depression, at its worst, can result in suicide. Regarding the degree and sequence of depressive episodes in a time, medical care providers may recommend psychotherapy in addition to various drugs, such as selective serotonin reuptake inhibitors (SSRIs), tricyclic antidepressants (TCAs), and tetracyclic antidepressants (Rouini et al., 2014; WHO, 2021). Mirtazapine (MRT) is an unconventional antidepressant authorized to treat moderate to severe depression patients, usually accompanied by anxiety disorders. It is a tetracyclic antidepressant that works on noradrenergic and specific serotonergic receptors (NaSSA). It is the only tetracyclic antidepressant licensed for the treatment of depression by the food and drug administration (FDA) (Rouini et al., 2014). Mirtazapine shows poor water solubility, with a partition coefficient of 2.9 (K. M. Ezealisiji et al., 2017). Additionally, it has a low bioavailability of about 50%. It was estimated that increasing its water solubility might result in increased bioavailability and reduce the dose required to achieve the desired therapeutic effect (K. E. Ezealisiji et al., 2015).

Recently, mesoporous silica nanostructures (MSNs) were introduced as platforms for boosting the apparent solubility and dissolution rate of a variety of pharmacologically active compounds. According to considerable physiochemical, toxicological, safety, and epidemiological evidence, these compounds offer no environmental or health hazards since they are biodegradable and biocompatible (Paper et al., 2014). Mesoporous silica has been approved by the FDA as a safe drug carrier, to be used as oral delivery ingredients in amounts up to 1500 mg per day (Bharti et al., 2015; Gonçalves, 2018). Likewise, the small particle size of MSNs reduces any possibility of toxic effects, as the smaller particles have higher mobility and longer circulation half-life. To form a nontoxic, effective, and reproducible biomedical MSNs system, monosized distribution is required, usually between 10 − 200 nm (Gonçalves, 2018). The main benefit of mesoporous silica for improving the water solubility of insoluble pharmaceuticals is its unique ordered structure, high surface area, large pore volume, tunable pore size, and simplicity of surface functionalization. MSNs offer a defined pore size at the nano-scale for the entrapment of drug molecules, enabling for decreasing the drug particle sizes to the nanosize, resulting in enhanced solubility. Additionally, the chosen drug can be loaded into these pores in an amorphous form (Jambhrunkar et al., 2014; Maleki et al., 2017). Compared to plain crystalline particles, the nano-scales amorphous drugs entrapped to mesoporous silica carriers have a lower lattice energy, leading to significantly improving the drug's dissolution features (Ren et al., 2020). Furthermore, the amorphous form of the molecules has been stabilized by the restricted area inside the pores, preventing long-range ordering, nucleation, or recrystallization of the encapsulated drug molecules (Abd-Elrahman et al., 2016).

The goal of this research was to investigate the optimum condition to load the Mirtazapine into MSNs using Box-Behnken Design (BBD) to achieve the optimal formula of Mirtazapine with desirable physicochemical features and enhanced oral bioavailability.

## 2. Materials and methods

### 2.1. Materials

Mirtazapine was kindly provided by Mash Premiere for Pharmaceutical Industry (New Cairo City, Egypt). Mesoporous silica nanostructures (SBA-15, MCM-41, and Aluminate-MCM-41) were purchased from XFNANO Materials Tech Co., Ltd (Beijing, CHINA). Absolute ethanol (99%) was obtained from (ADWIC, El-Nasr Pharmaceutical Chemicals Co, Cairo, Egypt). All chemicals and reagents were HPLC grade.

### 2.2. Methods

#### 2.2.1. Experimental design

A Box-Behnken Design with three factors at three levels (3^3^) was developed to study the effect of the independent variables on the dependent responses through response surface analysis. The experimental design and statistical analysis of BBD were achieved using the Design-Expert^®^ software trial (Kim et al., 2021). The design demanded to construct 17 trials (Table 2). The three examined variables were the drug/MSNs ratio (A), the type of MSNs (B), and the loading method (C), which was picked as independent variables after coding them (-1, 0, and 1), whereas the loading efficiency (Y_1_), aqueous solubility (Y_2_), and release after 30 min (Y_3_) were considered as dependent variables (Table 1). The software program creates one equation per response that shows the effect of the independent variables on this response. This equation can be individually applied for each response.

**Table 1.** Factores and responses with their values used by BBD.

Response Surface Design			
Factors	Low (-1)	Central (0)	High (+1)
A: (Drug/MSNs) ratio	33.33%	49.99%	66.66%
B: MSNs type	MCM-41	SBA-15	Aluminate-MCM-41 (Alu-MCM-41)
C: Loading method	Impregnation	Evaporation	Incipient
Responses	Goal		
Y_1_: Loading efficiency	Maximize		
Y_2_: Solubility	Maximize		
Y_3_: Release after 30 min	Target to 100 %		

**Table 2.** Summary of BBD.

Formula	Drug/MSNs ratio	MSNs type	Loading method	Loading efficiency (%)	Solubility (mg/ml)	Release after 30 min (%)
1	49.99	MCM-41	Impregnation	85.55	0.096	49.9
2	49.99	SBA-15	Evaporation	93.03	0.124	71.01
3	49.99	SBA-15	Evaporation	93.03	0.124	71.01
4	33.33	Alu-MCM-41	Evaporation	88.02	0.135	81.71
5	49.99	SBA-15	Evaporation	93.03	0.124	71.01
6	66.66	Alu-MCM-41	Evaporation	95.63	0.105	45.02
7	33.33	SBA-15	impregnation	96.19	0.137	80.12
8	66.66	SBA-15	impregnation	107.95	0.126	45.50
9	49.99	Alu-MCM-41	Incipient	95.23	0.113	25.20
10	33.33	SBA-15	Incipient	104.05	0.200	100
11	66.66	SBA-15	Incipient	105.08	0.133	65.60
12	49.99	MCM-41	Incipient	94.21	0.098	29.94
13	49.99	Alu-MCM-41	impregnation	106.36	0.111	22.86
14	49.99	SBA-15	Evaporation	93.03	0.124	71.01
15	49.99	SBA-15	Evaporation	93.03	0.124	71.01
16	66.66	MCM-41	Evaporation	98.01	0.0925	45.52
17	33.33	MCM-41	Evaporation	86.05	0.099	55.34

#### 2.2.2. Optimization of formulation components

The optimal MSNs formula selection was performed using the maximum desirability, which provided for simultaneous evaluation of all responses. The optimization step was established to achieve the maximum loading efficiency and solubility and to target the release rate after 30 min to 100%. The experiment with the highest degree of desirability (close to 1) was picked. Furthermore, when the probability (p-value) is far below 0.05, the surface response design is found to be significant (Albash et al., 2020).

#### 2.2.3. Mirtazapine loading into mesoporous silica nanostructures

Three different methods were studied for MRT loading into MSNs namely: Incipient wetness method, solvent evaporation method, and solvent impregnation method. The loading process was carried out by using the ethanol as a loading solvent. These methods were applied by using three different types of MSNs: SBA-15, MCM-41, and Alu-MCM-41; through different drug/MSNs ratios (33.33%), (49.99%) and (66.66%).

##### 2.2.3.1 Incipient wetness method

Ethanol solution containing MRT at a concentration of 100 mg/ml was injected drop-wise to the mesoporous materials; at various drug to carrier ratios. Following that, the capillary action fills the pores of MSNs. The mixture of MRT-MSNs was dried at 40 °C for 48 hours in a vacuum oven (Fisher Isotemp Oven 100 series, Model 127 G, USA) (Seljak et al., 2020).

##### 2.2.3.2 Solvent evaporation method

Mesoporous materials were introduced to an ethanol solution containing MRT at a concentration of 10 mg/ml; at various drug to carrier ratios. The mixture was agitated at 25 °C for 2 hours by a magnetic stirrer (Model MSH-20D, GmbH, Germany). After that, the ethanol in the solution was evaporated at 40 °C by using a rotatory evaporator (Heidolph rotavapor vv 2000/WB 2000, Germany). The prepared material was allowed to dry for 24 hours at room temperature (Soares, 2013).

##### 2.2.3.3 Solvent impregnation method

A concentrated ethanol solution of MRT (20 mg/ml) is immersed with mesoporous materials; at various drug-to-carrier ratios and agitated for 48 hours at 70 °C in a shaking water path (Lab – line, USA). Subsequently, the MRT-MSNs are obtained by air drying for 24 hours followed by 48 hours in vacuum drying at 40 °C to remove the whole solvent (Lai et al., 2017; Seljak et al., 2020).

#### 2.2.4. Determination of loading efficiency

The amount of drug-loaded was estimated and compared with the theoretical drug weight to ensure and validate the preparation method's efficiency. This test is carried out by dissolving a small amount (10 mg) of the prepared formula in 10 ml ethanol to prepare 1 mg/ml of nanostructure solution. One ml of this solution was centrifuged at 10,000 rpm for 10 min by using cooling centrifuge (3 K 30, Sigma, Germany), adequately diluted and evaluated by UV- Vis spectrophotometer (UV-1650P.C, Shimaduzu Corporation, Koyoto, Japan) at λ_max_ 293 nm (Pande et al., 2019).

(1) $$\text{Loading Efficiency \% }=\;\frac{\text{Practical weight of drug}}{\text{Theoretical weight of drug}}\text{ x }100$$

#### 2.2.5. Saturation aqueous solubility study

The plain MRT and the produced formulas have been subjected to saturated aqueous solubility assessment. An excess of the material to be examined was added to 5 ml distilled water in locked containers. The samples were agitated for 48 hours at 37 ± 1 °C in an incubator shaker. The solution was passed through a 0.45 μm Millipore filter, and the drug concentration was evaluated by UV- Vis spectrophotometer at λ_max_ 293 nm (K. E. Ezealisiji et al., 2015).

#### 2.2.6. *In-vitro* drug release study

The dissolution profiles of the plain MRT and the MRT-MSNs formulas were evaluated using USP standard dissolution apparatus II (Dr. Schleuniger Pharmatron AG, DIS 6000, Switzerland). Each dissolution experiment was conducted in triplicate. Each was weighed precisely and added to 500 mL of distilled water. The paddle was rotated at a speed of 50 rpm at a temperature of 37 ± 1 °C. Two milliliters of aliquot samples were collected and replaced to maintain the sink condition. Adequate dilution and subsequent filtration of samples were performed using 0.45 μm Millipore filters before measuring their absorbance by UV- Vis spectrophotometer at λ_max_ 293 nm (J. Patel et al., 2011).

To understand the release mechanism of MRT from MSNs, five models were applied, which are a zero-order model, first-order, the Higuchi release model, the Korsmeyer– Peppas, and the Weibull model. Correlation coefficients (R^2^) would be used to assess each model's degree of fit (Ibrahim et al., 2021).

#### 2.2.7. *In-vitro* characterization of optimized MSNs formula

##### 2.2.7.1 Gas adsorption manometry

The nitrogen adsorption and desorption analysis are the most effective methods for investigating the porosity of porous material and how the drug significantly changed this porosity. It was performed to obtain data about the surface area, pore size, and total pore volume of the SBA-15 and the optimized formula of MRT loaded into SBA-15 at 77k using Nitrogen Adsorption/Desorption analyzer (NOVA touch 2LX, USA). The surface area was measured using the Brunauer–Emmett–Teller (BET) method. Pore size and pore volume were determined using the Barrett-Joyner-Halenda (BJH) method (Soares, 2013; Budiman, 2019).

##### 2.2.7.2 Fourier-transform infrared spectroscopy (FT-IR)

Fourier-transform infrared spectrum (FTIR) analysis was performed to assess the possible interaction between MRT and MSNs, depending on the presence or absence of the characteristic MRT peaks, shifting and masking of MRT peaks due to loading into SBA-15, or the appearance of new peaks. FTIR of plain MRT, SBA-15, and the optimized formula of MRT-SBA-15 were obtained by using FTIR spectrophotometer (Shimadzu Affinity-1, Japan). The samples were combined with KBr, compacted into a disk, and evaluated with a resolution of 4 cm^−1^ over the range from 4000 cm^−1^ to 400 cm^−1^ (Madan et al., 2018 ).

##### 2.2.7.3 Differential scanning calorimetry (DSC)

The differential scanning calorimetry (DSC) test was used to investigate the thermal behavior of the drug in MSNs by using a differential scanning calorimeter (Q 600 SDT Simultaneous DSC, USA). DSC curves of plain MRT, SBA-15, optimized MRT-SBA-15, and a physical mixture of MRT and SBA-15 is obtained. The samples (about 2–4 mg) were fixed to aluminum pans and heated from 20 to 250 °C at a rate of 10 °C/min in a nitrogen atmosphere (El-Nabarawi et al., 2016).

##### 2.2.7.4 X-ray powder diffraction (XRPD)

The purpose of XRPD analysis is to investigate the crystalline nature of plain MRT. Samples of plain MRT, SBA-15, optimized MRT-SBA-15 and physical mixture of MRT and SBA-15 were filled into the holder and irradiated with monochromatized Cu Kα radiation at 30 kV and 30 mA. The step size within an angle of (2θ) over a range of 5°-50°, using the X-ray diffractometer (Scintage XDS 2000 diffractometer, USA) (Varshosaz et al., 2019).

##### 2.2.7.5 Polarized light microscopy (PLM)

Polarized light microscopy (PLM) is a method used for evaluating the morphology of the plain drug and the nanoparticle structure of the loaded silica. The polarized optical microscopy images of plain MRT and the optimized MRT-SBA-15 samples were obtained by the Polarized light microscope (polarized Nikon, Japan) under the same light conditions (Budiman, 2019).

##### 2.2.7.6 Scanning electron microscopy (SEM)

Scanning electron microscopy (SEM) was conducted to assess the morphology of the mesoporous silica before and after drug loading by using the Scanning electron microscope (JSM-6360, Japan). The samples were gold-coated prior to examination. About 1 mg of each sample was adhered to a sample holder using a double-sided sticky strip. SEM images were recorded at an accelerating voltage of 15 (Le et al., 2019).

#### 2.2.8. High performance liquid chromatography (HPLC) study

##### 2.2.8.1 HPLC analysis of rabbits’ plasma samples

The bioanalytical method based on HPLC was used to separate and analyze MTZ in rabbits' plasma. The mobile phase was comprised of phosphate buffer (pH 3.9) and acetonitrile (90:10). The flow rate was 1 ml/min. At ambient temperature, samples were injected into column C18 Xterra (4.6x100mm, 5 µm) by isocratic elution. A photodiode array detector (Waters 996 photodiode, USA) was used to achieve the detecting wavelength of 293 nm. The experiments were carried out using Empower 2 software on the HPLC system (Waters 2690 Alliance HPLC, USA) (Ibrahim et al., 2021).

##### 2.2.8.2 Standard curve of Mirtazapine in rabbits’ plasma

A stock solution of MRT in methanol (1 mg/ml) was prepared. It was serially diluted by spiking blank rabbits’ plasma with a suitable amount of stock solution to attain (200, 400, 600, 800, and 1000 ng/ml) to construct a calibration curve. Mix 200 μl from each concentration with 1 ml acetonitrile for 2 min using a vortex mixer (Cryste-Novapro, Bucheon, Korea), then centrifuge the mixture at 15,000 rpm for 15 min with a cooling centrifuge. The supernatant was vaporized under a nitrogen stream, then the mobile phase (120 μl) used to reconstitute it and passed through a 0.22 m syringe filter to be filtered. The sample (100 μl) was fed into the HPLC apparatus for examination (Ibrahim et al., 2021).

#### 2.2.9. *In-vivo* study of optimized MSNs formula

Ten healthy adult white rabbits weighing 1600–1800 gm were supplied by the Zagazig University's animal unit, Egypt, and housed under (12 hour) light/dark cycle, at room temperature. They were starved for 24 hours before the trial and maintained fasted for 6 hours following drug administration with free access to water (K. M. Ezealisiji et al., 2017). The investigation performed followed the regulations of the Guide for the Care and Use of Laboratory Animals (National Research Council, 2011) and the guide of Zagazig University, Faculty of Pharmacy, Institutional Animal Care and Use Committee (IACUC) (Approval number: ZU-IACUC/3/F/105/2021).

##### 2.2.9.1 Determination of MRT concentration in rabbit plasma

A single-blinded randomized study was conducted to test the drug bioavailability. Ten rabbits were randomly divided into two groups. Group I received plain MRT after being suspended in water, group II was given MRT loaded into SBA-15 (optimized formula). Administration was orally by pharyngostomy tube (4 French) and dose equal to 15 mg/kg (El-Sisi et al., 2017; Salazar-Juárez et al., 2017). Orbital blood samples (0.5 ml) were collected at 0, 0.5, 1, 2, 3, 5, 8, 11, and 24 hours in a heparinized tube, centrifuged at 4000 rpm for 10 min to separate plasma. Plasma was kept at − 20 °C until required. The concentration of MRT in each plasma sample was calculated using HPLC at λ_max_ of 293 nm (K. M. Ezealisiji et al., 2017).

##### 2.2.9.2 Calculation of pharmacokinetic parameters

The principal parameters such as maximum plasma concentration (C_max_), the time required to reach this concentration (T_max_), the area under the plasma concentration-time curve (AUC_0-∞_) and (AUC_0-t_), and t_1/2_ was calculated using (PK solver program). The relative bioavailability of the optimized MRT-SBA-15 was calculated and compared to the oral suspension of plain MRT using the following equation (Albash et al., 2019):

(2) $$\text{Relative bioavailability }(\%)=({\mathrm{AUC}}_{0‒t}\;(\text{optimized MRT}-\mathrm{SBA}-15)/{\mathrm{AUC}}_{0‒t}(\mathrm{Control}))\times 100.\;$$

## 3. Results and discussion

### 3.1. Experimental data analysis and validation

The Box Behnken design was employed to evaluate the independent variables (Drug/MSNs ratio (A), MSNs type (B), and Loading method (C)), optimizing and assessing the process's principal impacts, interaction effects, and also the quadratic effect on the dependent variables (loading efficiency, aqueous solubility and release after 30 min). Based on BBD, the design proposes seventeen tests for the response surface methodology. Table 2 illustrates the experimental design, in which diverse factors resulted in distinct responses. The findings clearly reveal that all dependent responses are highly related to the chosen independent variables, as shown by the presence of a significant P-value (Table 3). The data setwas studied using (ANOVA) by Design Expert^®^ software, which was used to obtain an analysis of variance, regression equation, and regression coefficients. The equations illustrate the quantitative effect of variables and their interaction on the responses (Y_1_, Y_2_, and Y_3_) (Venugopal et al., 2016).

**Table 3.** Anova results of the quadratic model for loading efficiency (Y_1_), solubulity(Y_2_), and release after 30 min (Y_3_).

	Model	R^2^	Adjusted R^2^	Predicted R^2^	Adequate precision	P-Value	F-ratio	
Y_1_	Quadratic	0.89	0.76	−0.65	10.23	0.0099	6.74	significant
Y_2_	Quadratic	0.91	0.81	−0.31	12.31	0.0047	8.69	significant
Y_3_	Quadratic	0.87	0.72	−0.94	7.74	0.0165	5.63	significant

#### 3.1.1. Effect of independent variables on loading efficiency (Y_1_)

**Figure 1.**
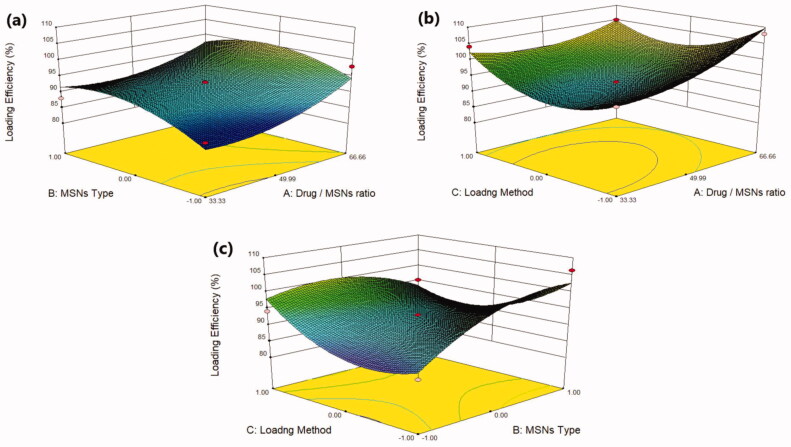
Response 3 D plots for the effect of (a) drug/MSNs ratio and MSNs type on loading efficiency (b) drug/MSNs ratio and loading method on loading efficiency (c) MSNs type and loading method on loading efficiency.

To load the drug effectively into the pore of MSNs, the pore size usually need to be larger than the drug molecule dimensions (Trzeciak et al., 2021). Mirtazapine was efficiently loaded into SBA-15, MCM-41, and Aluminite-MCM-41, as they have a suitable pore size of 3.28 nm, 1.69 nm, and 1.68 nm respectively, which are larger than the size of the drug molecule. In general, the pore diameter: drug molecule size should be >1 (Chaudhari and Gupte, 2017). Furthermore, the negatively charged SiO^-^ groups present on the surface of these types of silica permit the electrostatic interactions with positively charged MRT molecules, leading to a greater loading capacity (Huang et al., 2014). The MRT was loaded into MSNs by Incipient wetness method, solvent evaporation method, and solvent impregnation method using the ethanol as a loading solvent, due to the high solubility of MRT in ethanol, allowing the drug molecules to enter the pore structure efficiently and uniformly in an appropriate time (Lehto & Riikonen, 2014). After drug loading, the solvent has been removed to the acceptable levels specified in the guidelines of the International Conference on Harmonization (ICH) Q3 (R5) to confirm the removal of all traces of organic solvent (Trzeciak et al., 2021). The loading efficiency for all formulas was in the range of 85.55% (F1) to 107.95% (F8) (Table 2). The effect of the principle and interactive variables on the loading efficiency was interpreted by 3 D response surface plot (Figure 1) and the polynomial Equation (3):

$$\text{Loading Efficiency}=+\;93.03+4.04*A+2.68*B+0.32\text{ * C}-1.09\text{*A*B}-2.68\text{*A*C}-4.95\text{*B*C}+3.44*A^{2}-4.54\;*B^{2}+6.85*C^{2}$$

According to the regression equation, the drug/MSNs ratio has a positive effect on the loading efficiency; when the drug ratio increases, the drug loading efficiency significantly increases. The Mirtazapine loaded into MSNs by the ratio of 66.66% showed the highest loading efficiency, whereas those loaded by 33.33% showed the lowest loading efficiency (Figure 1); this might be due to the fact that increasing the drug feeding ratio leads to more drugs could be incorporated into the MSNs resulted in increased drug loading (Palanikumar et al., 2015). The MSNs pore size and surface chemistry have a considerable effect on the drug loading efficiency. The drug loading efficiency increased when loaded into MSNs by the following order: MCM-41 < Alu-MCM-41< SBA-15 (Figure 1). The SBA-15 showed the largest pore size of 3.28 nm, while MCM-41 and Alu-MCM-41 had a comparable pore size of 1.69 nm and 1.68 nm, respectively. This would indicate that the nanostructures with greater pore size had a higher loading efficiency. This could be due to the distribution of MRT within the SBA-15 mesopores, which are wider than MCM-41 and Alu-MCM-41 pores, allowing for easier and more uniform drug loading (Li, Zhang and Feng, 2019). Moreover, while Alu-MCM-41 and MCM-41 have similar pore sizes, Alu-MCM-41 seems to have a higher loading efficiency. This is caused by functionalizing and modifying the siliceous frameworks through the incorporation of aluminum metal ions in the silica mesopores; this leads to increase the loading efficiency by constructing coordination interactions between the metal species and the drug moieties (Kankala et al., 2020). All three drug loading procedures were successful and beneficial in loading the MRT into MSNs; readily available methods can be used even if they are not optimal for the intended purpose (Lehto & Riikonen, 2014). Although the solvent impregnation and solvent evaporation methods showed higher loading efficiency (Figure 1), the incipient wetness method considered the optimal method for loading MRT into SBA-15; as it has a high drug concentration in the initial loading solution, driving the drug molecules more efficiently to enter the pores of the material and stays trapped within the pores after the solvent was removed. Furthermore, the high efficiency of this method may be due to the high solubility of MRT in ethanol, which is a low viscosity loading solvent, permitting the MRT molecules to be located inside the micropores of the mesoporous walls and deposited along the pore walls, ensuring that no drug is left to crystallize on the surface (Mccarthy et al., 2016).

#### 3.1.2. Effect of independent variables on aqueous solubility (Y_2_)

**Figure 2.**
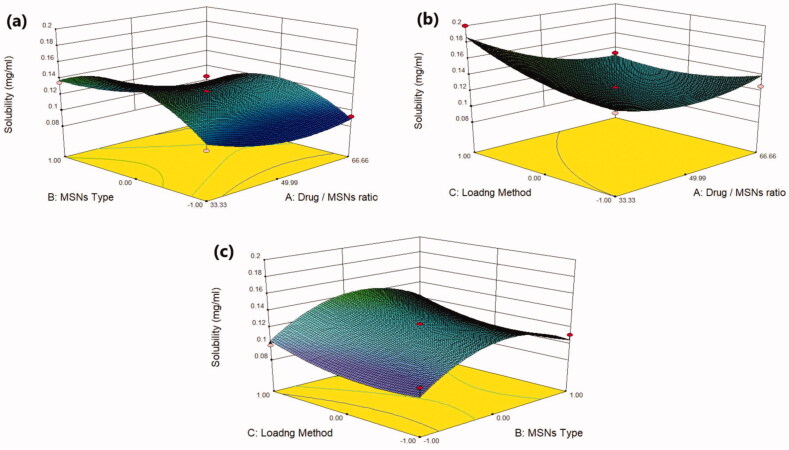
Response 3 D plots for the effect of (a) drug/MSNs ratio and MSNs type on solubility (b) drug/MSNs ratio and loading method on solubility (c) MSNs type and loading method on solubility.

Plain MRT demonstrates a poor aqueous solubility of 0.092 mg/ml, whereas all MRT-loaded samples showed improvement in MRT solubility but with varying degrees (Table 2). The effect of the principle and interactive variables on the loading efficiency was interpreted by 3 D response surface plot (Figure 2) and the polynomial Equation (4):

$$\mathrm{Solubility}=+\;0.12-0.014*A+9.813*B+9.25*C-5.875\text{*A*B}-0.014\text{*A*C}+0.000\text{*B*C}+0.014*A^{2}\;-0.030*B^{2}+0.011*C^{2}$$

According to the polynomial equation, the saturation aqueous solubility is affected negatively by the drug/MSNs ratio, whereas decreasing the drug ratio leads to an increase in the aqueous solubility of MRT. The Mirtazapine-MSNs ratio of 66.66% showed the lowest aqueous solubility, whereas the loading ratio of 33.33% showed the highest aqueous solubility (Varshosaz et al., 2019) (Figure 2). This is assumed to the fact that most drugs changed from crystalline to amorphous state with lower lattice energy after being encapsulated into MSNs with a low drug ratio, resulting in increased drug solubility (T. Li et al., 2019). As the drug to MSNs ratio increases, surface adsorption of drug particles with nearly crystalline form may occur, resulting in a decrease in MRT solubility at these high drug ratios (Chand et al., 2019). The aqueous solubility of MRT is influenced by the pore volume and particle size of the MSNs. The MSNs have a pore volume of 1.42 cc/g, 1.01 cc/g and 0.52 cc/g for SBA-15, MCM-41 and Alu-MCM-41, respectively. The greatest increase in solubility was observed with those loaded into SBA-15 (largest pore volume); this could be attributed to MRT's complete amorphous state and increased active surface area after encapsulation into SBA-15 (Abd-Elbary et al., 2014). Accordingly, the solubility of MRT was increased when loaded into MSNs in the sequence of: MCM-41 < Alu-MCM-41< SBA-15 (Figure 2). The average particle size of MRT loaded into SBA-15, MCM-41, and Alu-MCM-41 were 55.34 nm, 166.3 nm, and 113.1 nm, respectively. According to the Kelvin equation, reducing the particle size led to increasing the aqueous solubility, and when the size of the particles is smaller than 100 nm, it would be perfect for boosting the saturation solubility due to an increase of dissolution pressure (Chand et al., 2019). The method used for loading MRT into MSNs can affect its aqueous solubility. The drug loaded by the incipient wetness method showed higher solubility than that loaded by rotary evaporation or solvent impregnation. In the incipient wetness method, the MRT molecules were found alongside the mesopore's walls and inside the micropores. The drug molecules in the micropores are restricted to such an extent that crystal growth may almost be inhibited (Mccarthy et al., 2016) (Figure 2).

#### 3.1.3. Effect of independent variables on release after 30 min (Y_3_)

**Figure 3.**
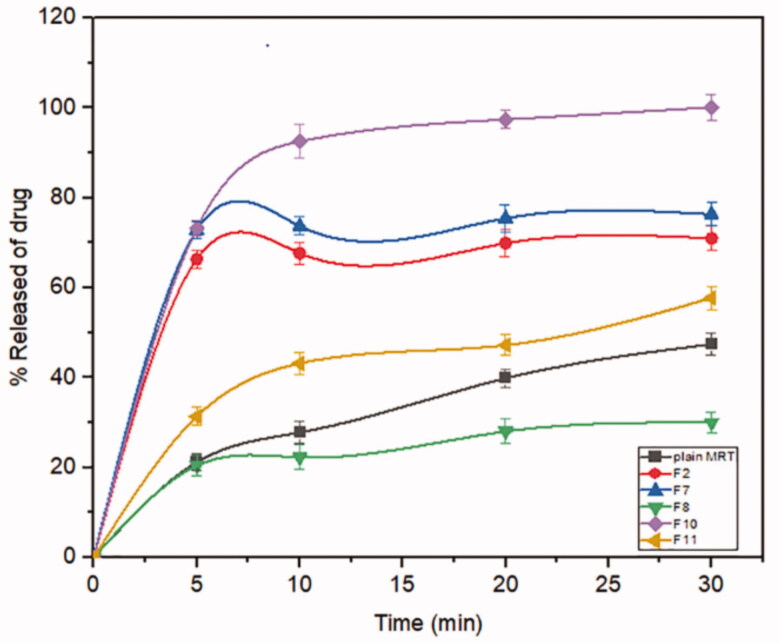
In-vitro release profiles of Mirtazapine-loaded into SBA-15.

**Figure 4.**
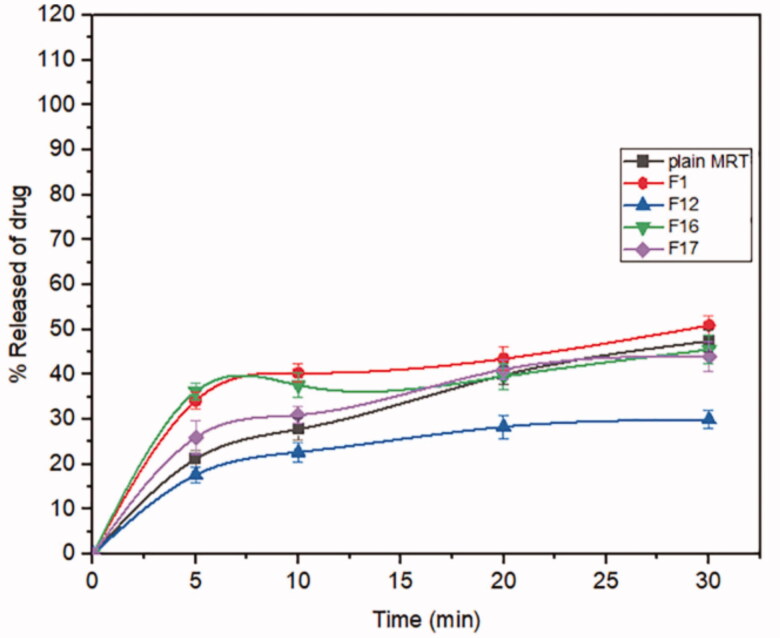
In-vitro release profiles of Mirtazapine-loaded into MCM-41.

**Figure 5.**
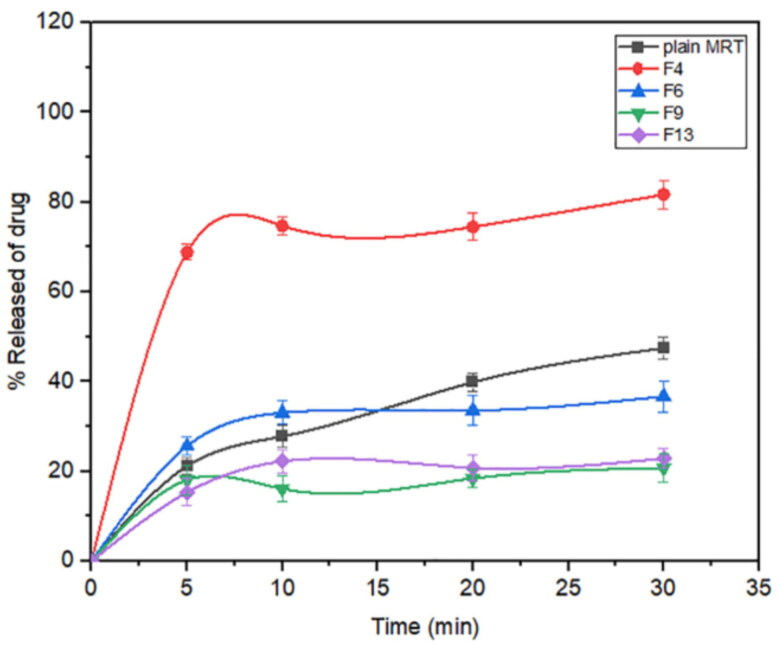
In-vitro release profiles of Mirtazapine -loaded into Alu-MCM-41.

**Figure 6.**
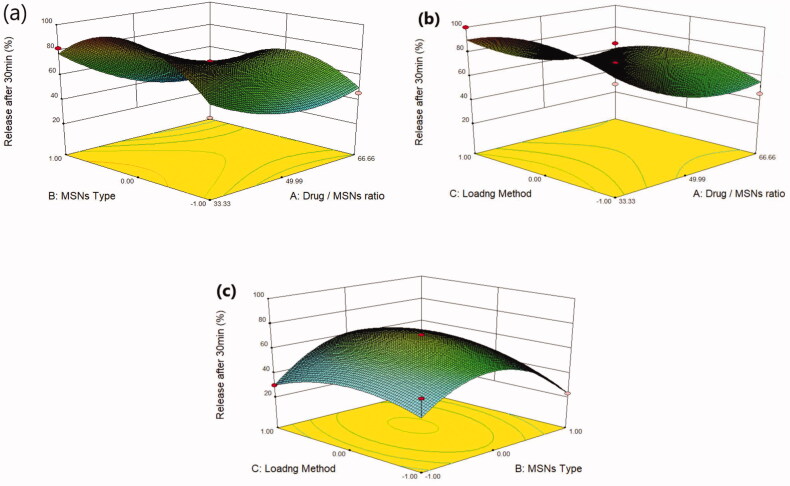
Response 3 D plots for the effect of (a) drug/MSNs ratio and MSNs type on release after 30 min (b) drug/MSNs ratio and loading method on release after 30 min (c) MSNs type and loading method on release after 30 min.

Mirtazapine's release profile from MSNs is biphasic, with a first burst release followed by a subsequent slow release, as illustrated in (Figure 3–5). The drug molecule can be entrapped physically to the MSNs, depending on the silica and drug molecule affinities, that are achieved primarily by weak physical interactions, resulting in a fast initial burst release. The second step has a slower release rate due to the presence of chemical bonds between the remaining drug and the silica's silanol groups (Kankala et al., 2019; Fiani et al., 2015). Furthermore, it has also been suggested that drug molecules closest to the surface release more rapidly due to their closeness to the dissolving media, whereas the deeper remnant in the pores release much more slowly (Mccarthy et al., 2016). The release of the drug from the host mesoporous may occur through passive diffusion of the drug through the pore channels (Gonçalves, 2018). The release profile of MRT after 30 min differed within the range of 22.86% (F13) to 100% (F10) (Table 2). The effect of the principle and interactive variables on the loading efficiency was elucidated by 3 D response surface plot (Figure 6) and the polynomial Equation (5):

$$\text{Release after }30\min\;=+\;71.0\;1\;-14.44\text{*A }-\;0.72\text{*B }+\;2.79\text{*C }-\;6.72\text{*A*B }+\;0.055\text{*A*C }+\;5.57\text{*B*C }+\;13.36*A^{2}-27.47*B^{2}-11.56*C^{2}$$

According to the equation, the drug ratio is the determining factor for drug release rate, which has a highly negative influence on the rate of MRT release from the MSNs. It was noticed that the MRT release rate from SBA-15, MCM-41, and Alu-MCM-41 decreased when loaded by the ratio of 66.66%, whereas those loaded by 33.33% showed the highest releaserate (Figure 6). The high release rate at low drug ratio is due to the drug's amorphous form, which dissolves quickly in the aqueous medium, bursting the MRT release profile. This can be accomplished by surface interactions between drugs and carriers while reducing the crystalline drug deposited on the outer surface of the particles, as surface coverage of hydrophobic drug leads to decreasing its wetting effects (Maleki et al., 2017). The dissolution rate of the loaded drug into MSNs can also be influenced by the silica pore size and pore geometry (Pande et al., 2019). The drug release rates have been shown to increase when loaded into MSNs in the given sequence: MCM-41 < Alu-MCM-41< SBA-15 (Figure 6). This can be clarified as SBA-15 has a larger pore size, so more dissolution fluid can penetrate pore channels, solubilizing the drug and diffusing its molecules into the liquid media, according to Maleki et al., 2017. In addition, it can also be explained by the pore geometry of SBA-15, which allows sufficient space in the middle of the pore for the drug molecules to readily diffuse. It is also noteworthy that the Si-OH groups in SBA-15 exist only at the silica surface, forming a weak hydrogen bond with the active compound that can be broken easily, enhancing the drug release rate. Loading MRT by the incipient wetness into SBA-15 showed a higher release rate than the other methods (Figure 6); as this technique loads the drug into the micropores of the mesoporous wall, favoring the largest pore volume of SBA-15 (Mccarthy et al., 2016).

**Figure 7.**
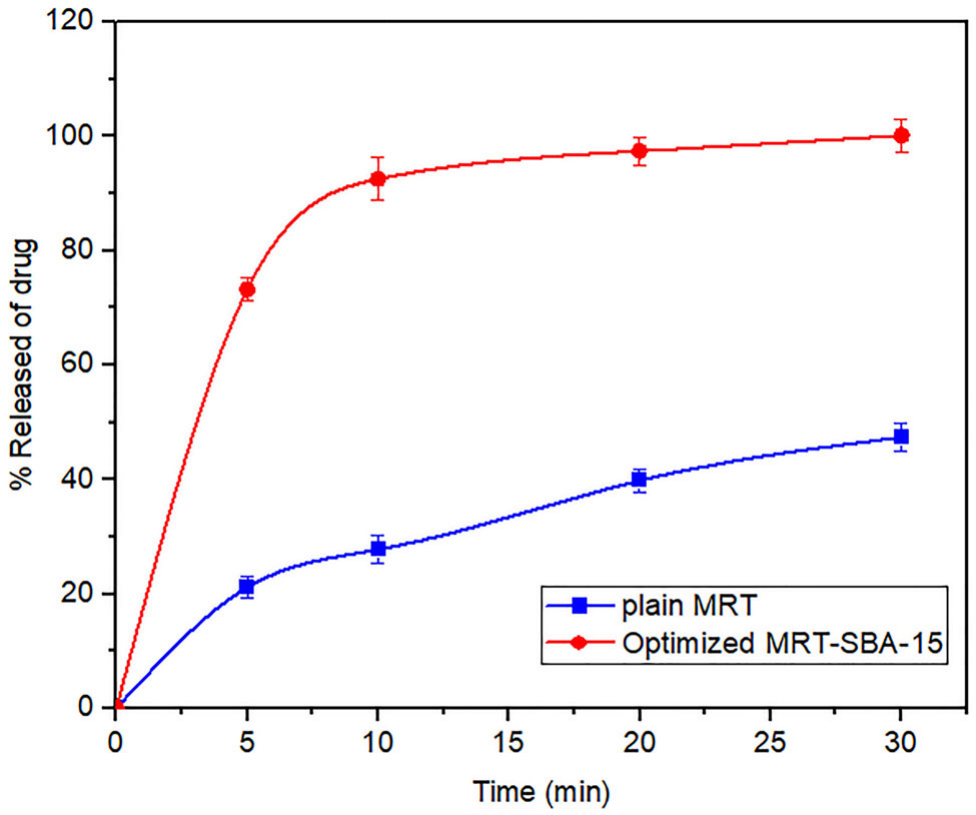
In-vitro release profiles of the optimized formula of Mirtazapine-SBA-15 and plain Mirtazapine.

The dissolution profiles of plain MRT and the optimal formula of MRT loaded into SBA-15 are shown in (Figure 7). It was obvious that the amorphous form of MRT inside the SBA-15 pores dissolve much more rapidly than the simple crystalline MRT. The release rate of MRT from SBA-15 was extremely rapid during the first 5 min, with nearly 75% of MRT dissolved. The maximum percentage of plain MRT dissolved was approximately 45% after 30 min, whereas the highest proportion of MRT dissolved and released from loaded SBA-15 was 100% at the same time. A comparison of dissolution profiles was performed since the dissolution rate of the optimized formula, and plain MRT within 15 min was less than 85% (Abd-Elrahman et al., 2016). Thus, the loaded MRT's difference factor f1 and similarity factor f2 were calculated in comparison to the plain MRT by using (DDSolver program). We found the values of f1 and f2 were 161.29 and 12.50, respectively, indicating a great difference in their dissolution profiles (Abd-Elrahman et al., 2016).

To identify the model that most accurately represented the drug's release pattern, we investigated in-vitro release data for plain MRT and the optimized MRT-SBA-15 using mathematical modeling equations. The optimized formula displayed a first-order release pattern with an R^2^ value of (0.9994). Additionally, it showed the highest R^2^ value for the Weibull model (0.9999). On the other hand, the plain MRT presented a first-order release pattern with an R^2^ value of (0.8580) and also showed a high R^2^ value of (0.9998) for the Korsmeyer-Peppas model (Abd-Elrahman et al., 2016; Ibrahim et al., 2021).

### 3.2. Optimization and validation of variables

Optimized situations were achieved by putting constraints on the dependent and independent variables. Optimization was conducted to attain the values of Drug/MSNs (A), Type of MSNs (B), and loading method (C), which maximize loading efficiency (Y_1_), aqueous solubility (Y_2_) and reach 100% release after 30 min (Y_3_) (Table 1) (Ibrahim et al., 2020). To verify these results, three batches of nanostructures were constructed in accordance with the BBD's predicted level. After the optimized MSNs were prepared and characterized, the experimental values for the desired responses were 104.05% for loading efficiency, 0.2 mg/ml for solubility, and 100% for release after 30 min. As illustrated in Table 4, both predicted and experimental results were consistent, demonstrating the rationality and validity of BBD results. Also, the residuals between the expected and actual responses were minimal, indicating the optimization step's reliability (Albash et al., 2020).

**Table 4.** Predicted and observed values of the optimaized MRT loaded into SBA-15.

Factor (independent variables)	Optimized level		
A: drug/MSNs (%)	33.33%		
B: type of MSN's	SBA-15		
C: loading method	Incipient wetness		
Responses (dependent variables)	Expected	Observed	Residual*
Y_1_: loading efficiency (%)	102.03	104.05	− 2.02
Y_2_: aqueous solubility (mg/ml)	0.188	0.200	− 0.012
Y_3_: release after 30 min (%)	91.02	100	−8.98

* Residual: expected-observed.

### 3.3. Characterization of the optimized formula

#### 3.3.1. Gas adsorption manometry

**Figure 8.**
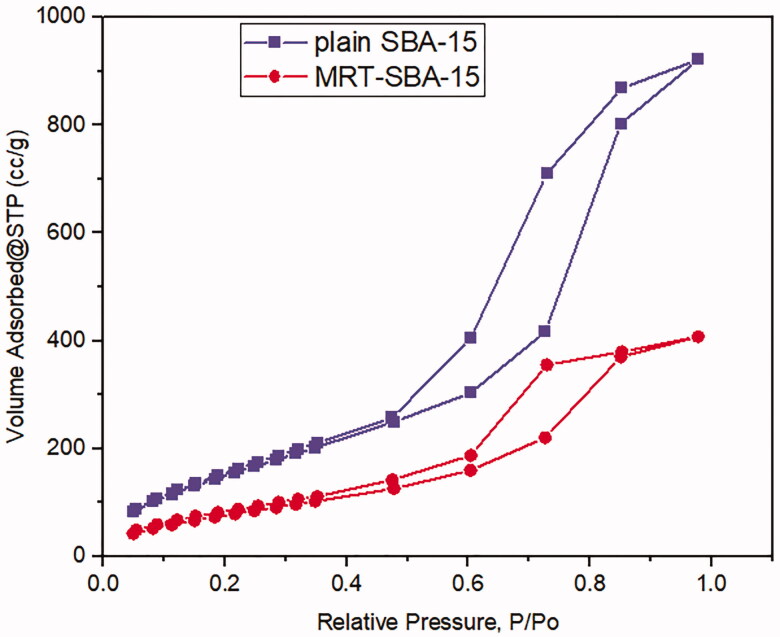
Ads/des isotherms of (a) plain SBA-15 (b) optimized MRT-SBA-15.

**Figure 9.**
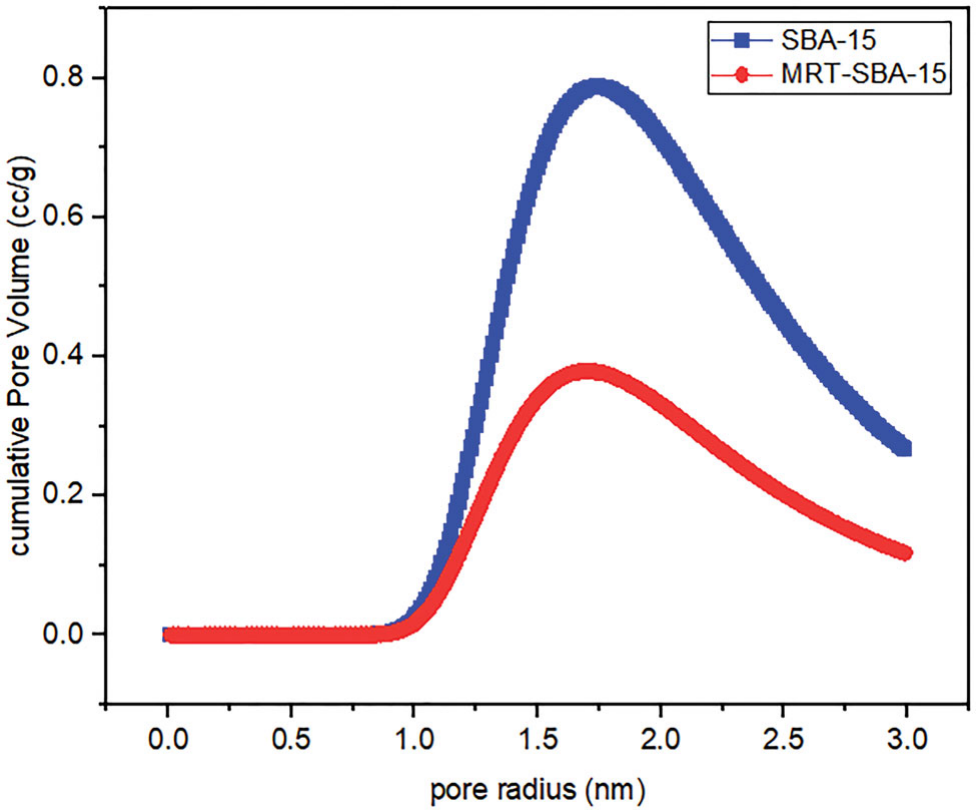
Pore size distribution of (a) plain SBA-15 (b) optimized MRT-SBA-15.

Nitrogen ads/des isotherms of plain SBA-15 exhibit a standard type IV curve with an H1 hysteresis loop according to IUPAC classification (Figure 8), which is associated with porous materials consisting of a well-defined cylindrical like pore channel (Figure 14). The Nitrogen ads/des isotherms also show reversible nitrogen condensation steps, demonstrating uniform mesopore architecture (T. Li et al., 2019). After drug loading into SBA-15, the pattern of isotherm stayed unchanged (Figure 8); this indicated that after MRT loading into the molecular sieves of SBA-15, the characteristic mesoporous channel structure did not destroy and still exist, which also verified by the uniformed pore size distribution curve depicted in (Figure 9) (Abd-Elbary et al., 2014). Reduction occurs in surface area and pore volume compared to plain SBA-15 (Table 5). This decrease indicated that MRT was efficaciously loaded into the SBA-15 pores, which have a substantial effect on the MRT's *in-vitro* and *in-vivo* behavior (Soares, 2013).

**Table 5.** Structural and textural parameters of plain MRT and optimized MRT loaded into SBA-15.

Material	Specific surface area (m^2^/g)	Pore size (nm)	Pore volume (cc/g)
SBA-15	621.13 m²/g	3.28 nm	1.42 cc/g
MRT-SBA-15	313.69 m²/g	3.28 nm	0.63 cc/g

#### 3.3.2. Fourier-transform infrared spectroscopy (FT-IR)

**Figure 10.**
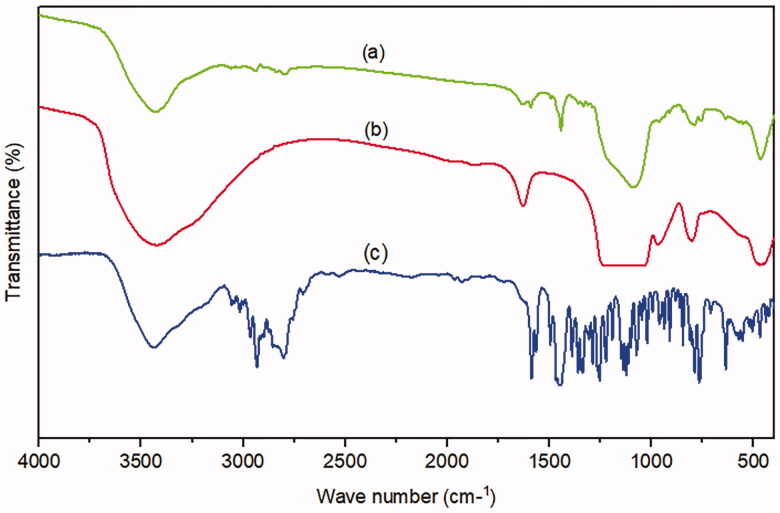
FT-IR of (a) optimized MRT-SBA-15 (b) plain SBA-15 (c) plain MRT.

Plain MRT (Figure 10(c)) showed its characteristic sharp peaks at 3439 cm^−1^ relatives to N-H stretching, a band at 2931 cm^−1^ originating from methyl group attached to N_2_ atom, and bands for C-C stretching of the phenyl group seemed at 1587 cm^−1^, and 1450 cm^−1^. The primary aromatic amines with N directly attached to the ring give bands at 1336 cm^−1^, and 1253 cm ^−1^. The benzene ring C-H appears in the ranges of 1359 cm^−1^ to 1074 cm^−1^ (Hamed and Hussein, 2020). Plain SBA-15 (Figure 10(b)) shows a major characteristic intense peak around 3439 cm^−1^, and a weak peak at 970 cm^−1^ represents Si-OH stretching and bending vibrations, respectively. The band at 1627 cm^−1^ was due to the carboxyl group (C–O–C) stretching vibration. The broad band around 1080 cm^−1^ could be due to asymmetrical stretching vibrations of Si-O-Si, overlapped with Si-O-C, C-O-C, and Si-C bond vibrations. The band around 804 cm^−1^ was found to be associated with symmetrical stretching vibrations of the Si-O-Si bond, whereas the bands at 468 cm^−1^, and 449 cm^−1^ could be associated with the Si-O-Si bond bending vibrations (Kokunešoski et al., 2010; Abd-Elbary et al., 2014). The optimized formula of MRT-SBA-15 (Figure 10(a)) revealed distinctive peaks associated with MSNs, whereas the characteristic peaks of plain MRT are absent. The peaks originating from the SBA-15 revealed some changes by decreasing the peaks at 3439 cm^−1^,1080 cm^−1^ and 468 cm^−1^ to less intense peaks and disappearing the peaks at 1627 cm^−1^, 970 cm^−1^, 804 cm^−1^, and 449 cm^−1^. In addition, a new peak was revealed at about 1442 cm ^−1^. These results showed that the isolated terminal silanol groups present in SBA-15 interact significantly with MRT functional groups, which is beneficial for achieving high drug loading content with no traces of drug molecules being adsorbed to the surface (Soares, 2013).

#### 3.3.3. Differential scanning calorimetry (DSC)

**Figure 11.**
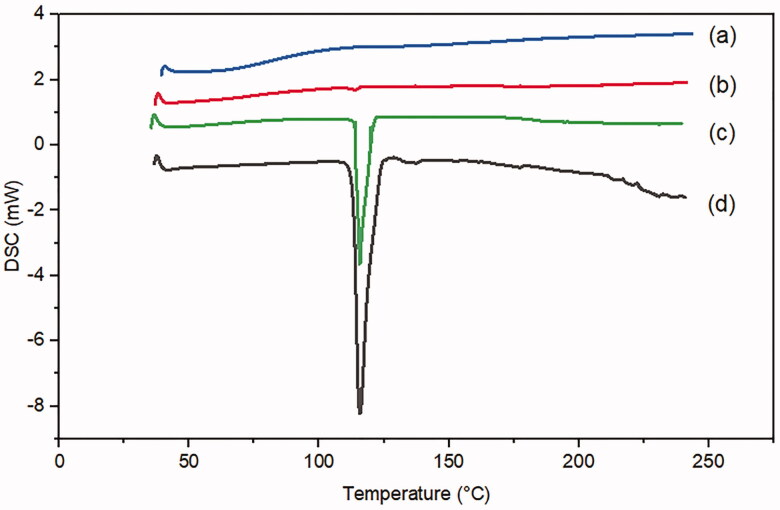
DSC curves of (a) plain SBA-15 (b) optimized MRT-SBA-15 (C) physical mixture of MRT/SBA-15(d) plain MRT.

A DSC analysis was conducted to investigate the MRT entrapment in the pores of the SBA-15, confirming that the loaded drug was present in amorphous form. The DSC spectrum of the plain MRT (Figure 11(d)) has a prominent sharp endothermic peak at 115 °C, demonstrating the MRT's crystalline nature. The curve of plain SBA-15 (Figure 11(a)) showed no peak, revealing that SBA-15 has an amorphous nature. However, the signal of crystalline MRT also can be detected in its corresponding physical mixtures (Figure 11(c)). In contrast to figures d and c, the optimized formula of MRT loaded into SBA-15 in (Figure 11(b)) revealed no evidence of the crystalline structure of MRT (Huang et al., 2019).

#### 3.3.4 X-Ray powder diffraction (XRPD)

**Figure 12.**
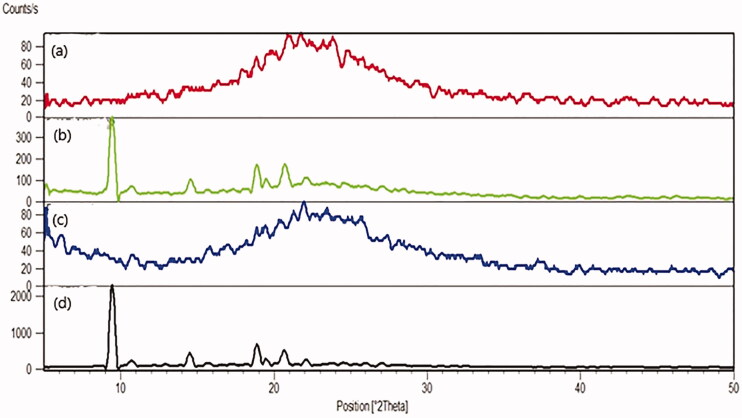
PXRD curves of (a) optimized MRT-SBA-15 (b) physical mixture of MRT/ SBA-15 (C) plain SBA-15 (d) plain MRT.

Mirtazapine is loaded effectively into the pores of SBA-15 when the main diffraction peaks disappear. The plain MRT exhibited distinct and strong diffraction peaks appearing at 2θ values of 9.5°, 14.5°, 19.0°, and 20.7°, which were suggestive of the MRT's extremely crystalline nature (Figure 12(d)). Additionally, the same peaks were detected when MRT and SBA-15 were physically mixed (Figure 12(b)). In contrast to the previous findings, plain SBA-15 (Figure 12(c)) did not display any prominent diffraction peaks because of their amorphous nature. Following the incorporation of MRT into SBA-15 (Figure 12(a)), no crystalline MRT was found in the MRT-SBA-15 XRPD pattern, indicating that MRT was loaded into the carrier in an amorphous state. It was assumed that when MRT was incorporated into the pores of SBA-15, crystallization was hindered due to the limitation of space, which rendered the MRT in a disordered amorphous condition (Zhang et al., 2018).This result in a complete agreement with the DSC result.

#### 3.3.5. Polarized light microscopy (PLM)

**Figure 13.**
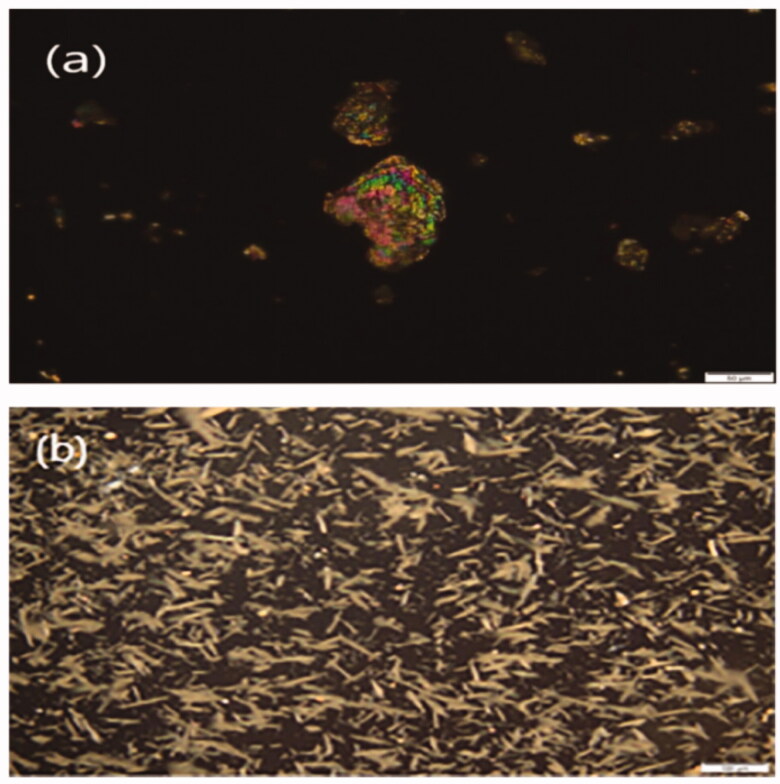
Polarized light microscopy (PLM) images of (a) plain MRT (b) optimized MRT- SBA-15.

The morphology of the plain MRT and optimized formula of MRT loaded into SBA-15 showed in the PLM images (Figure 13). The plain MRT was exposed as brilliant crystals when viewed by polarized light. After loading of MRT into SBA-15, the sample presented a wide size distribution of regular rod-shaped particles. The morphology of the drug incorporated into SBA-15 is completely different from the plain drug, indicating the absence of plain MRT crystals in the optimized formula (Lai et al., 2017; Budiman, 2019).

#### 3.3.6. Scanning electron microscopy (SEM)

**Figure 14.**
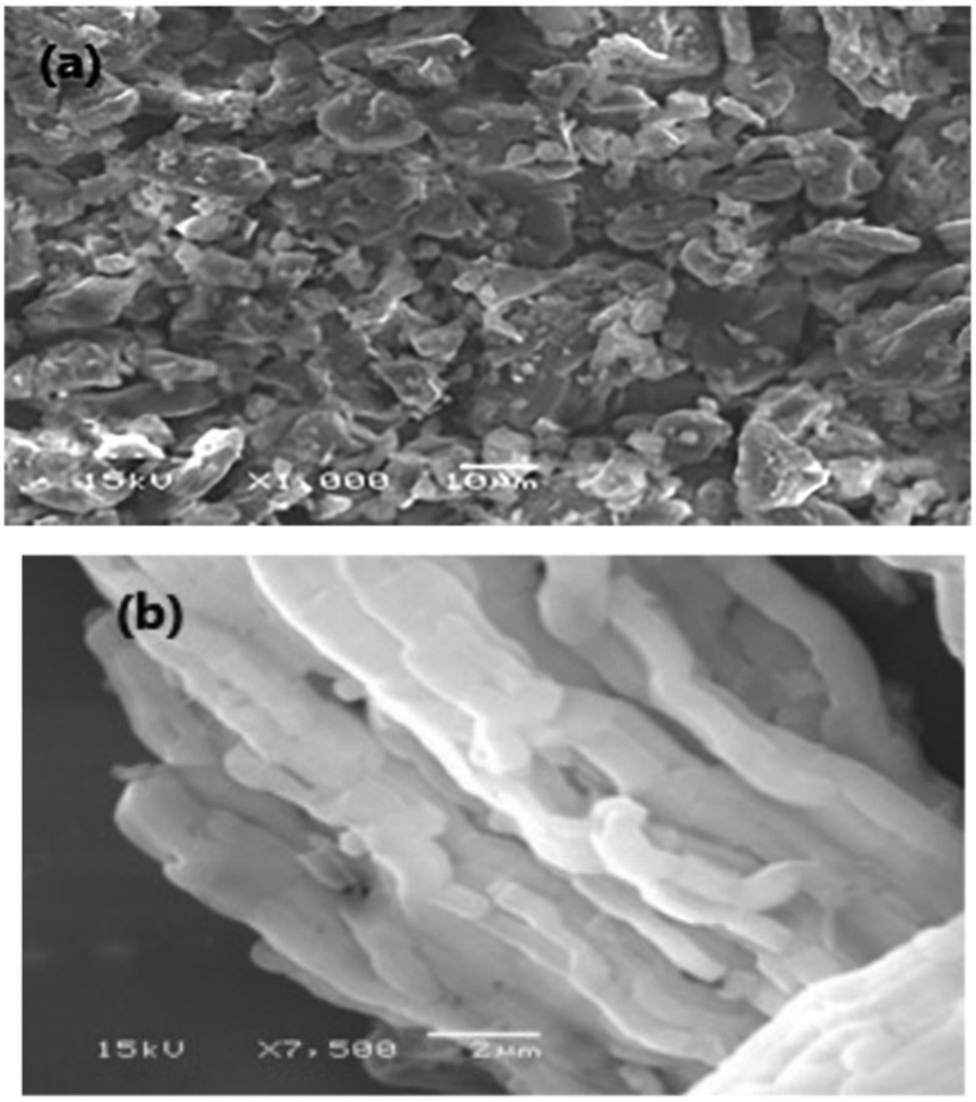
SEM images of (a) plain MRT(x1000) (b) optimized MRT-SBA-15 (X 7,500).

SEM in (Figure 14) investigates the surface morphology of plain MRT and optimized formula of MRT-loaded into SBA-15, using a high-resolution technique. The image shows the crystalline nature of plain MRT, which disappeared when loaded into SBA-15, revealing an amorphous form of MRT arranged in a well-ordered array bundle of longitudinal- rod-shaped SBA-15 (Zhai, 2020). This change was the principal cause for the rapid dissolution and fast onset of action of the loaded MRT-SBA-15. The SEM shows a similar result with the PLM result.

### 3.4. *In-vivo* pharmacokinetic studies on rabbit plasma samples

**Figure 15.**
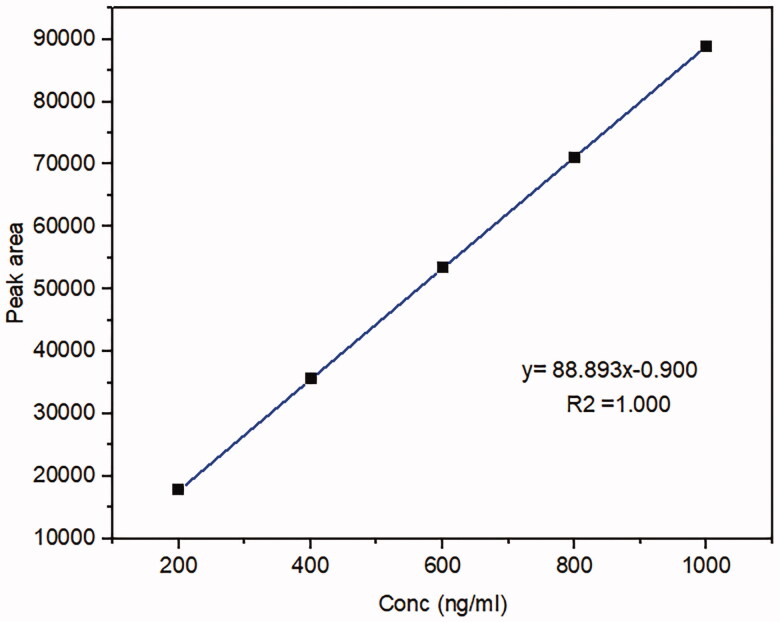
HPLC standard curve of Mirtazapine in rabbit’s plasma.

**Figure 16.**
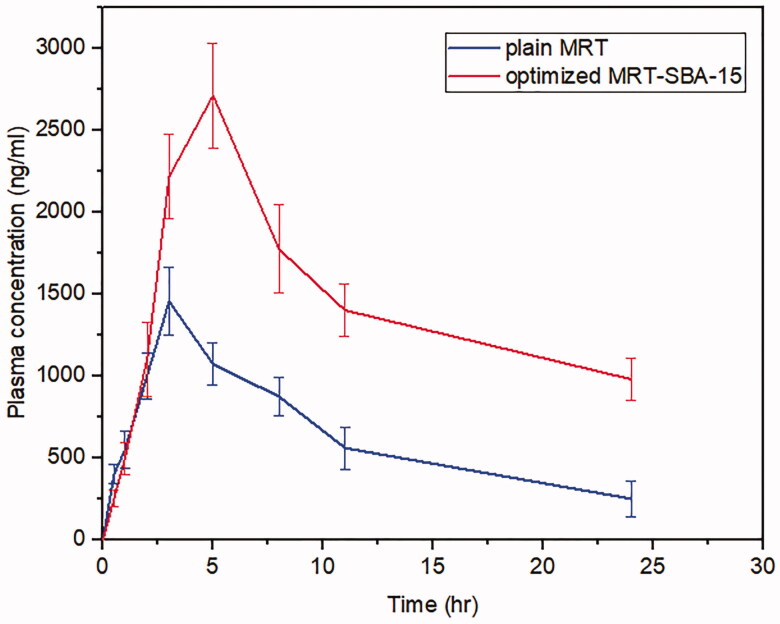
Plasma levels of Mirtazapine after administration of plain MRT oral suspension and optimized MRT-SBA-15.

The bioavailability investigation was determined in rabbits' plasma by using the HPLC method to evaluate whether the MSNs had a significant impact on improving drug oral bioavailability. Firstly, The HPLC technique was established and validated to determine the MRT concentrations in rabbits' plasma quantitatively. A calibration curve was constructed by graphing the peak area (Y) against the drug concentration (X) in the range of 200 to 1000 ng/ml (Figure 15). The standard plasma curve shows good linearity with an R^2^ value of 1 (R. B. Patel, 2017). The mean plasma concentrations as a function of time after oral administration of the optimized MRT-SBA-15 formula and plain MRT are shown in Figure 16. The main pharmacokinetic parameters are displayed in Table 6. According to Student's t-test, the increase in the C_max_ value of the optimized formula is statically significant (*p* < 0.05). Additionally, the exposure AUC_0-t_ for the optimized formula was significantly higher than that of the plain drug (*p* < 0.05). The optimized MRT-SBA-15 formula shows a tremendous increase in oral bioavailability, an approximately 2.14-fold increase than plain MRT. Accordingly, from these findings we speculate this formula might show a good bioavailability when used orally in humans. As a result, the optimized formula indicates that a lower therapeutic dosage might be used to obtain an equivalent clinical benefit with fewer side effects (Wang et al., 2012). Notably, this work proved the capacity of porous silica to overcome a drug's low solubility and boost its oral bioavailability.

**Table 6.** Pharmacokinetic parameters of MRT oral suspension and optimized MRT-SBA-15.

Pharmacokinetic parameters	Oral suspension	Optimized MRT-SBA-15
C_max_ (ng/mL)	1455.71 ± 210.85	2918.22 ± 320.62
T_max (_hr)	3	5
t _½_ (hr)	3.88 ± 0.23	6.04 ± 0.58
AUC_0-t (_ng/ml.hr)	14192.06 ± 340.83	30448.72 ± 985.76
AUC_0-∞_ (ng/ml.hr)	14483.73 ± 1088.15	33066.49 ± 1368.34
Relative bioavailability (%)	–	214.54 %

C_max_: maximum plasma concentration; T_max_: time required to reach maximum plasma concentration; t_1/2_: elimination half-life; AUC_0-t_: area under the plasma concentration-time curve; AUC_0-∞:_ area under the curve from time zero to infinity.

## 4. Conclusion

Mirtazapine was successfully encapsulated into SBA-15, MCM-41, and Aluminite-MCM-41. The loading process was carried out using different loading procedures through different drug/MSNs ratios. The Box Behnken design was chosen to evaluate how the independent variables influence the dependent responses to optimize the MRT loading efficiency, solubility and releasing performance. Additionally, the optimized formula was subjected to morphological and physiochemical assessment, which indicated that MRT was entrapped efficiently into the pores of SBA-15 in its amorphous state. Finally, the *in vivo* study demonstrated an increase in the oral bioavailability of the optimized MRT loaded into SBA-15 by 2.14-fold compared to the plain drug in the rabbit's plasma. Further studies are recommended to incorporate this newly developed formula into a suitable oral dosage form and measure its *in-vivo* bioavailability. As a result, the findings demonstrated that the optimal response was obtained by loading MRT into SBA-15 by using of the incipient wetness method at a drug to silica ratio of 33.33%, which successfully eliminated the medication's extensive poor water solubility and boosted its oral bioavailability.

## References

[CIT0002] Abd-Elrahman AA, El Nabarawi MA, Hassan DH, Taha AA. (2016). Ketoprofen mesoporous silica nanoparticles SBA-15 hard gelatin capsules: preparation and in vitro/in vivo characterization. Drug Deliv 23: 1582–98. 10.1080/10717544.2016.1186251 27167529

[CIT0003] Albash R, El-Nabarawi MA, Refai H, Abdelbary AA. (2019). Tailoring of PEGylated bilosomes for promoting the transdermal delivery of olmesartan medoxomil: in-vitro characterization, ex-vivo permeation and in-vivo assessment. Int J Nanomedicine 14:6555–74. 31616143 10.2147/IJN.S213613 PMC6699521

[CIT0004] Albash R, Elmahboub Y, Baraka K, et al. (2020). Ultra-deformable liposomes containing terpenes (terpesomes) loaded fenticonazole nitrate for treatment of vaginal candidiasis: Box-Behnken design optimization, comparative ex vivo and in vivo studies. Drug Deliv 27: 1514–23. 33108907 10.1080/10717544.2020.1837295 PMC7594706

[CIT0005] Bharti C, Nagaich U, Kumar Pal A, Gulati N. (2015). Mesoporous silica nanoparticles in target drug delivery system: a review. Int J Pharm Investig 5:124–33. 10.4103/2230-973X.160844. PMC4522861 26258053

[CIT0006] Budiman A. (2019). Characterization of drugs encapsulated into mesoporous silica. Int J App Pharm 11: 7–11.

[CIT0007] Chand YY, Pattnaik S, Swain K. (2019). Curcumin loaded mesoporous silica nanoparticles: assessment of bioavailability and cardioprotective effect. Drug Dev Ind Pharm 45: 1889–95. 31549866 10.1080/03639045.2019.1672717

[CIT0008] Chaudhari SP, Gupte A. (2017). Mesoporous silica as a carrier for amorphous solid dispersion. BJPR 16: 1–19.

[CIT0009] El-Nabarawi MA, Khalil IA, Saad RM. (2016). Impact of hydrophilic polymer solubilization on bioavailability enhancement of repaglinide by solid dispersion. Inventi Rapid: Pharm Tech 2016: 1–12.

[CIT0010] El-Sisi AE, El-Sayad ME, Abdelsalam NM. (2017). Protective effects of mirtazapine and chrysin on experimentally induced testicular damage in rats. Biomed Pharmacother 95: 1059–66. 28922723 10.1016/j.biopha.2017.09.022

[CIT0011] Ezealisiji KE, Mbah CJ, Osadebe PO. (2015). Aqueous solubility enhancement of mirtazapine: effect of cosolvent and surfactant. Pharmacol Pharmacy 06: 471–6.

[CIT0012] Ezealisiji KM, Mbah CJ, Osadebe P, Krause R. (2017). Pharmacokinetics studies of mirtazapine loaded nanoemulsion and its evaluation as transdermal delivery system. J Chem Pharmaceutical Res 9: 74–84.

[CIT0013] Fiani E, Vierling M, Molie M. (2015). Comptes rendus chimie synthesis of CuO/SBA-15 adsorbents for SO x removal applications, using different impregnation methods ` Se d ’ Adsorbants CuO/SBA-15 Par Diffe ’. Rentes Me. 18:1013–29.

[CIT0014] Gonçalves MC. (2018). Sol-gel silica nanoparticles in medicine: a natural choice. design, synthesis and products. Molecules 23: 2021–6. 30104542 10.3390/molecules23082021 PMC6222648

[CIT0015] Hamed HE, Hussein AA. (2020). Preparation, in vitro and ex-vivo evaluation of mirtazapine nanosuspension and nanoparticles incorporated in orodispersible tablets. IJPS 29: 62–75.

[CIT0016] Huang X, Young NP, Townley HE. (2014). Characterization and comparison of mesoporous silica particles for optimized drug delivery. Nanomater Nanotechnol 4: 2–15.

[CIT0017] Huang Y, Zhao X, Zu Y, et al. (2019). Enhanced solubility and bioavailability of apigenin via preparation of solid dispersions of mesoporous silica nanoparticles. Iran J Pharm Res 18: 168–82. 10.22037/ijpr.2019.2347. 31089353 PMC6487423

[CIT0018] Ibrahim TM, Eissa RG, El-Megrab NA, El-Nahas HM. (2021). Morphological characterization of optimized risperidone-loaded in-situ gel forming implants with pharmacokinetic and behavioral assessments in rats. J Drug Delivery Sci Technol 61:102195.

[CIT0019] Ibrahim TM, El-Megrab NA, El-Nahas HM. (2020). Optimization of injectable PLGA in-situ forming implants of anti-psychotic risperidone via box-behnken design. J Drug Delivery Sci Technol 58:101803.

[CIT0020] Jambhrunkar S, Qu Z, Popat A, et al. (2014). Modulating in vitro release and solubility of griseofulvin using functionalized mesoporous silica nanoparticles. J Colloid Interface Sci 434:218–25. 25203914 10.1016/j.jcis.2014.08.019

[CIT0021] Kankala RK, Ya Hui Han J, Na Chia Hung Lee Z et al. (2020). Nanoarchitectured structure and surface biofunctionality of mesoporous silica nanoparticles. Adv Mater 32: 1907035–27. 10.1002/adma.201907035 32319133

[CIT0022] Kankala RK, Zhang H, Liu CG, et al. (2019). Metal species–encapsulated mesoporous silica nanoparticles: current advancements and latest breakthroughs. Adv Funct Mater 29: 1902652–42.

[CIT0023] Kim MK, Ki DH, Na YG, et al. (2021). Optimization of mesoporous silica nanoparticles through statistical design of experiment and the application for the anticancer drug. Pharmaceutics 13:184. 33572523 10.3390/pharmaceutics13020184 PMC7911876

[CIT0024] Kokunešoski M, Gulicovski J, Matović B, et al. (2010). Synthesis and surface characterization of ordered mesoporous silica SBA-15. Mater Chem Phys 124: 1248–52.

[CIT0025] Lai J, Lin W, Scholes P, Li M. (2017). Investigating the effects of loading factors on the in vitro pharmaceutical performance of mesoporous materials as drug carriers for ibuprofen. Materials 10:150. 28772509 10.3390/ma10020150 PMC5459193

[CIT0026] Le T-t, Khaliq A, Elyafi E, et al. 2019. Delivery of poorly soluble drugs via mesoporous silica: impact of drug overloading on release and thermal profiles. Pharmaceutics. 11:269. 31185610 10.3390/pharmaceutics11060269 PMC6630575

[CIT0027] Lehto VP, and Riikonen J. (2014). “14 - Drug Loading and Characterization of Porous Silicon Materials”. In: Hélder A. Santos, editor. Porous Silicon for Biomedical Applications. Amsterdam: Woodhead Publishing, 337–355.

[CIT0028] Li T, Geng T, Md A, et al. (2019). Novel Scheme for Rapid Synthesis of Hollow Mesoporous Silica Nanoparticles (HMSNs) and their application as an efficient delivery carrier for oral bioavailability improvement of poorly water-soluble BCS Type II drugs. Colloids Surf B Biointerfaces 176:185–93. 30616109 10.1016/j.colsurfb.2019.01.004

[CIT0029] Li Z, Zhang Y, Feng N. (2019). Mesoporous silica nanoparticles: synthesis, classification, drug loading, pharmacokinetics, biocompatibility, and application in drug delivery. Expert Opin Drug Deliv 16: 219–37. 30686075 10.1080/17425247.2019.1575806

[CIT1001] Madan JR, Patil S, Mathure D, et al. (2018). Improving dissolution profile of poorly water-soluble drug u s i n g non-ordered mesoporous silica. Marmara Pharmaceutical Journal 22: 249–58. 10.12991/mpj.2018.62.

[CIT0031] Maleki A, Kettiger H, Schoubben A, et al. (2017). Mesoporous silica materials: From physico-chemical properties to enhanced dissolution of poorly water-soluble drugs. J Control Release 262:329–47. 28778479 10.1016/j.jconrel.2017.07.047

[CIT0032] Mccarthy CA, RJ, Ahern R, Dontireddy KB, Ryan, et al. 2016. Mesoporous silica formulation strategies for drug dissolution enhancement: a review mesoporous silica formulation strategies for drug dissolution enhancement: a review. Expert Opin Drug Deliv 13: 93–108. 26549623 10.1517/17425247.2016.1100165

[CIT0033] National Research Council. 2011. Guide laboratory animals for the care and use of laboratory animals. 8th ed. NW Washington (DC): The National Academies Press, 246.

[CIT0034] Palanikumar L, Choi ES, Cheon JY, et al. (2015). Noncovalent polymer-gatekeeper in mesoporous silica nanoparticles as a targeted drug delivery platform. Adv Funct Mater 25: 957–65.

[CIT0035] Pande VV, Jadhav KS, Giri MA, et al. (2019). Design and development of paliperidone mesoporous silica template as a platform for surge dose drug delivery system. Mater Technol 34: 117–25.

[CIT0036] Paper R, Huang X, Young NP, Townley HE. (2014). Characterization and comparison of mesoporous silica particles for optimized drug delivery regular paper. Nanomater Nanotechnol 4:2–15.

[CIT0037] Patel J, Kevin G, Patel A, et al. (2011). Design and development of a self-nanoemulsifying drug delivery system for telmisartan for oral drug delivery. Int J Pharm Investig 1: 112–8. 10.4103/2230-973X.82431 PMC3465127 23071930

[CIT0038] Patel RB, Patel MR, Mehta JB. (2017). Validation of stability indicating high performance liquid chromatographic method for estimation of desloratadine in tablet formulation. Arabian J Chem 10:S644–S50.

[CIT0039] Nemeroff CB, Schatzberg AF, Rasgon N, Strakowski SM. The American Psychiatric Association Publishing textbook of mood disorders. Washington, DC: American Psychiatric Association Publishing; 2022.

[CIT0040] Ren X, Cheng S, Liang Y, et al. (2020). Mesoporous silica nanospheres as nanocarriers for poorly soluble drug itraconazole with high loading capacity and enhanced bioavailability. MicroporousMesoporous Mater 305:110389–8.

[CIT0041] Rouini MR, Lavasani H, Sheikholeslami B, et al. (2014). Pharmacokinetics of mirtazapine and its main metabolites after single intravenous and oral administrations in rats at two dose rates. Daru 22:13. 10.1186/2008-2231-22-13. 24397986 PMC3896718

[CIT0042] Salazar-Juárez A, Barbosa-Méndez S, Merino-Reyes P, et al. (2017). Chronic dosing with mirtazapine does not produce sedation in rats. Braz J Psychiatry 39: 228–36. 28355345 10.1590/1516-4446-2016-2058 PMC7111384

[CIT0043] Seljak KB, Kocbek P, Gašperlin M. (2020). Mesoporous silica nanoparticles as delivery carriers: an overview of drug loading techniques. J Drug Delivery Sci Technol 59:101906.

[CIT0044] Soares AP. (2013). Development of mesoporous silica nanoparticles of ritonavir with enhanced bioavailability potential: formulation optimization, in-vitro and in-vivo evaluation.. J Chem Inf Model 53: 1689–99.23800267

[CIT0045] Trzeciak K, A, Chotera-ouda II, Bak-sypien MJ. Potrzebowski 2021. Mesoporous silica particles as drug delivery systems — the state of the art in loading methods and the recent progress in analytical techniques for monitoring these processes. Pharmaceutics. 13:950. 34202794 10.3390/pharmaceutics13070950 PMC8309060

[CIT0046] Varshosaz J, Dayani L, Chegini SP, Minaiyan M. (2019). Production of a new platform based on fumed and mesoporous silica nanoparticles for enhanced solubility and oral bioavailability of raloxifene HCl. IET Nanobiotechnol 13: 392–9. 31171744 10.1049/iet-nbt.2018.5252 PMC8676563

[CIT0047] Venugopal V, Kumar KJ, Muralidharan S, et al. (2016). OpenNano optimization and in-vivo evaluation of isradipine nanoparticles using box-behnken design surface response methodology. OpenNano 1:1–15.

[CIT0048] Wang Z, Chen B, Quan G, et al. (2012). Increasing the oral bioavailability of poorly water-soluble carbamazepine using immediate-release pellets supported on SBA-15 mesoporous silica. Inter J Nanomed 7:5807–18. 10.2147/IJN.S37650 PMC3509994 23209366

[CIT0049] WHO. 2021. Depression. https://www.who.int/news-room/fact-sheets/detail/depression.

[CIT0050] Zhai QZ. (2020). Study on SBA-15 as an effective sorbent for dye butyl rhodamine B. J Sol-Gel Sci Technol 96: 34–46.

[CIT0051] Zhang W, Zheng N, Chen L, et al. (2018). Effect of Shape on mesoporous silica nanoparticles for oral delivery of indomethacin. Pharmaceutics 11:4. 30583601 10.3390/pharmaceutics11010004 PMC6359657

